# Disparities in glaucoma and macular degeneration healthcare utilization among persons living with dementia in the United States

**DOI:** 10.1007/s00417-024-06573-z

**Published:** 2024-07-12

**Authors:** Ali G. Hamedani, Angela Y. Chang, Yineng Chen, Brian L. VanderBeek

**Affiliations:** 1 Department of Neurology, Perelman School of Medicine, University of Pennsylvania, Philadelphia, PA USA; 2 Department of Ophthalmology, Perelman School of Medicine, University of Pennsylvania, Philadelphia, PA USA; 3 Department of Biostatistics, Epidemiology, and Informatics, Perelman School of Medicine, University of Pennsylvania, Philadelphia, PA USA; 4 Leonard Davis Institute of Health Economics, University of Pennsylvania, Philadelphia, PA USA; 5 Center for Preventive Ophthalmology and Biostatistics, Perelman School of Medicine, University of Pennsylvania, Philadelphia, PA USA

**Keywords:** Glaucoma, Macular degeneration, Dementia, Healthcare utilization, Disparities

## Abstract

**Purpose:**

Dementia is common among patients with primary open angle glaucoma (POAG) and neovascular age-related macular degeneration (nAMD). This study compares visit frequency, diagnostic test utilization, and treatment patterns for POAG and nAMD among persons with vs. without dementia.

**Methods:**

Optum’s de-identified Clinformatics^®^ Data Mart Database (January 1, 2000-June 30, 2022) was used for this study. Two cohorts were created from newly diagnosed POAG or nAMD patients. Within each cohort, an exposure cohort was created of newly diagnosed dementia patients. The primary outcome was the number of visits to an eye care provider. Secondary analyses for the POAG cohort assessed the number of visual field tests, optical coherence tomography (OCT), and glaucoma medication prescription coverage. The secondary analysis for the nAMD cohort included the number of injections performed. Poisson regression was used to determine the relative rates of outcomes.

**Results:**

POAG patients with dementia had reduced rates of eye care visits (RR 0.76, 95% CI: 0.75–0.77), lower rates of testing utilization for visual fields (RR 0.66, 95% CI: 0.63–0.68) and OCT (RR 0.67, 95% CI: 0.64–0.69), and a lower rate of glaucoma prescription medication coverage (RR 0.83, 95% CI: 0.83–0.83). nAMD patients with dementia had reduced rates of eye care visits (RR 0.74, 95% CI: 0.70–0.79) and received fewer intravitreal injections (RR 0.64, 95% CI: 0.58–0.69) than those without dementia.

**Conclusions:**

POAG and nAMD patients with dementia obtained less eye care and less monitoring and treatment of their disease. These findings suggest that this population may be vulnerable to gaps in ophthalmic care.

**Supplementary Information:**

The online version contains supplementary material available at 10.1007/s00417-024-06573-z.

## Key messages

***What is known***

● Older adults with dementia are less likely to see an ophthalmologist or receive cataract surgery, but treatment disparities have not been examined in the context of other eye diseases.

***What is new***

● Patients with primary open-angle glaucoma who have dementia are less likely to be seen by an ophthalmologist, have a visual field test or OCT, or have an active prescription for an intraocular pressure-lowering eyedrop than those without dementia.

● Neovascular age-related macular degeneration patients with dementia are also less likely to see an ophthalmologist or receive intravitreal injections than those without dementia.

● Persons living with dementia may be vulnerable to gaps in ophthalmic care.

**Supplementary Information:**

The online version contains supplementary material available at 10.1007/s00417-024-06573-z.

## Introduction

Approximately 7 million people currently live with dementia in the U.S [1]. , and because age is the primary risk factor for dementia, this number is expected to increase dramatically in the coming decades as the older adult population grows in size [2]. Age is also a risk factor for many common eye diseases: by age 80, one out of every 20 individuals has glaucoma, late-stage age-related macular degeneration (AMD), or both [3, 4]. As a result, dementia is a common comorbidity among older adults with macular degeneration and glaucoma.

Visual impairment is a known risk factor for cognitive decline and neuropsychiatric symptoms of dementia [5–7], so close monitoring and treatment of ophthalmic disease may have a role in improving functional outcomes and quality of life in these patients in addition to preserving vision. However, there are multiple challenges associated with treating eye disease in older adults with dementia. Patients require frequent visits with an ophthalmologist, especially when receiving intravitreal therapies for AMD, which may strain the limits of caregiver availability. Once patients transition to living in a nursing home, they are even less likely to continue receiving specialty care [8]. Neuropsychiatric symptoms of dementia such as agitation may make intraocular pressure-lowering eyedrops for glaucoma challenging to administer, and in the case of in-office or surgical procedures like intravitreal injections, certain treatments may not be possible if patients are unable to lie still. Even when treatments are possible, ophthalmologists may perceive that patients with functional impairment and reduced life expectancy are unlikely to benefit from them.

Several recent studies have found that persons living with dementia are less likely to see an ophthalmologist or receive cataract surgery compared to those without dementia [9–11]. However, these differences have not been examined in the context of other age-related eye diseases such as glaucoma or AMD, for which delays in diagnosis and treatment are associated with adverse visual outcomes [12]. In this study, we used administrative claims data to compare visit frequency, diagnostic test utilization, and treatment patterns for glaucoma and neovascular AMD among persons with vs. without dementia.

## Methods

### Database

Optum’s de-identified Clinformatics^®^ Data Mart Database was used to abstract data for this study. The database contains all outpatient medical claims (office visits, procedures, and medications given) as well as demographic data and some laboratory values for all patients enrolled in commercial and Medicare Advantage insurance plans. The subset of data available for this study included all patients in the database from January 1, 2000, to June 30, 2022. Due to the de-identified nature of the data, The University of Pennsylvania’s Institutional Review Board deemed this study exempt from review.

### Cohorts

All newly diagnosed patients with primary open angle glaucoma (POAG) or neovascular age-related macular degeneration (nAMD) were included in the study creating two cohorts, one for each disease state. (Please see Supplemental Table 1 for the full list of ICD-9/ICD-10 and CPT codes used during this study.) Patients in the POAG cohort were excluded for having a diagnosis of a non-POAG form of glaucoma at any time in the plan. Patients were excluded from the nAMD cohort having any other disease that could be treated with a vascular endothelial growth factor (VEGF)-directed agent. Within each of the two cohorts, an exposure cohort was created of patients who were concurrently diagnosed with dementia. The index date was considered the earliest date of dementia diagnosis. Additional exclusion occurred for any patient who had less than 1 year of time in the insurance plan before and after the index date. To better assess the impact the diagnosis of dementia had on the care of these patients, all patients who were diagnosed with dementia prior to the cohort disease state (POAG, nAMD) were also excluded.

Disease-specific controls were identified from each cohort and matched up to 3:1 with dementia cases based on age, race, sex, and insurance plan start and stop date (± 4 months). After matching occurred, controls were assigned the index date of their dementia-exposed match. All exclusion and inclusion criteria were then applied to the controls the same as the exposed cohort. A secondary analysis was also performed that defined dementia either by ICD code or a prescription of a memory-enhancing medication. Of note, while diagnosis codes have good positive predictive value (up to 70%) for identifying people living with dementia [13], the timing of diagnosis is much less reliable. Specifically, because of its insidious onset and known delays in diagnosis [14], the months or years prior to the first appearance of a diagnosis code are likely also characterized by the presence of dementia. For this reason, we chose to compare POAG and nAMD patients with dementia to those without dementia rather than examining healthcare utilization before vs. after dementia diagnosis.

### Outcomes

The primary outcome for both cohorts was the number of visits to an eye care provider in the year following the index date. Secondary analyses for the POAG cohort also included the number of visual field tests performed, OCT’s obtained, and the number of days of glaucoma prescription coverage over the year following the index date. The secondary analysis for the nAMD cohort included the number of injections performed over the following year.

### Statistical analysis and covariates

Poisson regression was used to determine the relative rates of outcomes as outlined above. Inverse probability treatment weighting (IPTW) was used to better balance baseline covariates between the dementia patients and matched controls. Covariates assessed included in the multivariable model included age, gender, race, history of diabetes mellitus, hypertension, hypercholesterolemia, kidney disease, stroke, ischemic heart disease, congestive heart failure, previous myocardial infarction, arrhythmia, peripheral artery disease, smoking, education level, household income, and geographic location. Missing values for demographic variables were categorized as “unknown”. To help adjust for the severity of glaucoma, any previous glaucoma surgeries were assessed as were the number of glaucoma medication classes used in the year prior to the index date. All statistical analyses were performed using SAS version 9.4 (SAS Institute Inc., Cary, NC) software. All tests were considered statistically significant at P values of 0.05 (two-tailed).

## Results

**Fig. 1:**
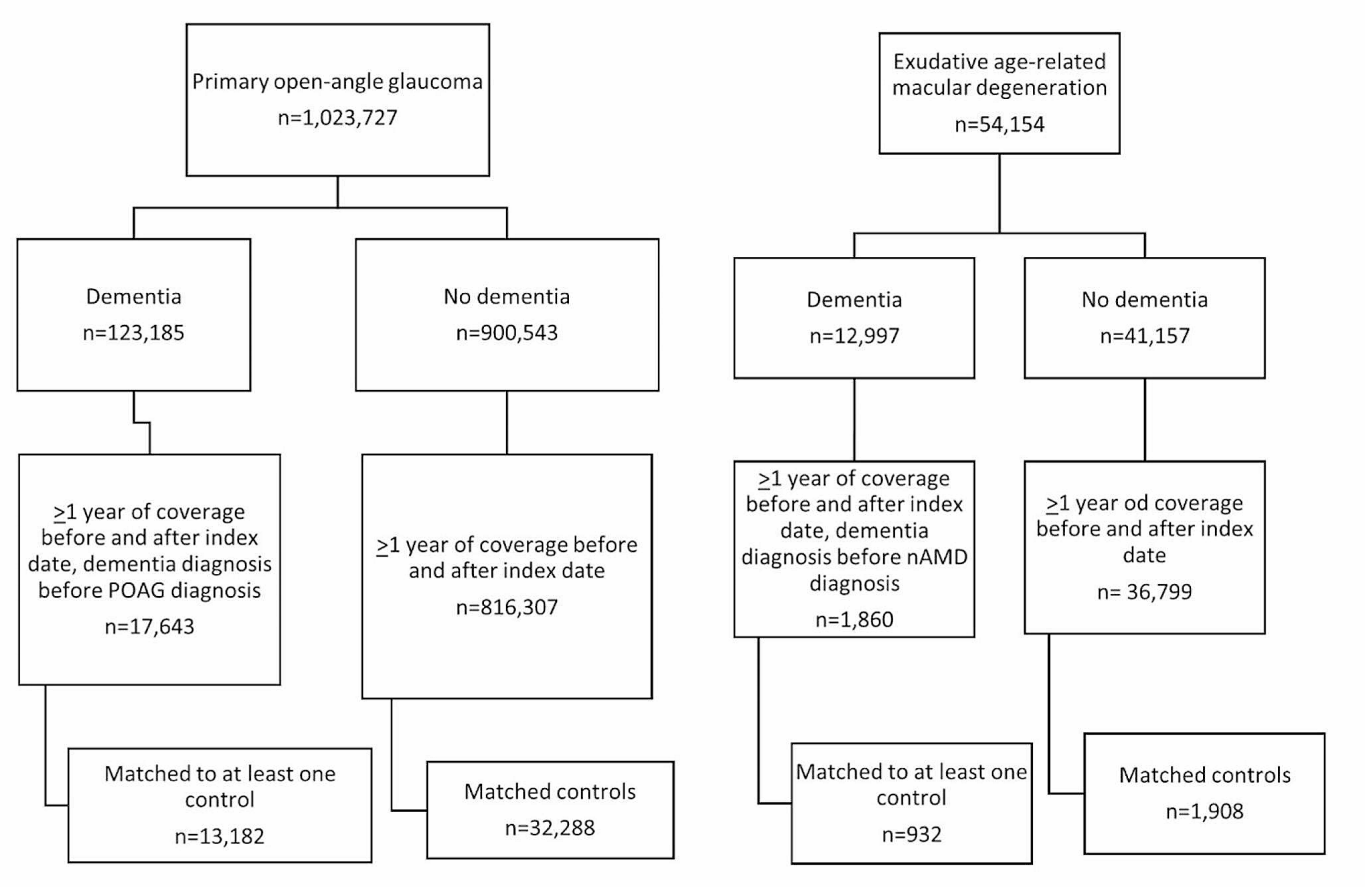
Summary of inclusion and exclusion criteria

We included 45,470 individuals with POAG (13,182 with dementia; 32,288 without) and 2,840 with nAMD (932 with dementia; 1908 without). Those included and excluded are summarized in Fig. 1, and demographic characteristics and comorbidities of the POAG and nAMD cohorts before and after inverse probability weighting are presented in Tables 1 and 2. Cases (dementia) and controls (no dementia) were generally well matched with excellent balance (standardized mean difference < 0.1) in both the POAG and nAMD cohorts after IPTW (Tables 1 and 2).

**Table 1:** Baseline characteristic of primary open-angle glaucoma patients with and without dementia

Comparison of baseline characteristics between cases and contols in POAG cohort									
Characteristic			Before Weighting			After Weighting			
			No Dementia (n = 32288)	Dementia (n = 13182)	Standardized mean difference	No Dementia (n = 32288)	Dementia (n = 13182)	Standardized mean difference	
Age [mean (SD)]		Mean (SD)	81.0 (6.9)	81.1 (6.8)	0.0168	81.1 (6.8)	81.2 (6.8)	0.0084	
Gender		Female	62%	62%	-0.0064	62%	62%	0.0010	
		Male	38%	38%		38%	38%		
Race		Asian	1%	2%	0.0838	1%	1%	0.0382	
		Black	11%	12%		11%	11%		
		Hispanic	7%	9%		8%	7%		
		White	80%	77%		79%	80%		
Education level		Bachelor Degree Plus	15%	16%	0.0551	15%	15%	0.0000	
		Less than Bachelor Degree	55%	55%		55%	56%		
		Less than or equal to HS Diploma	29%	28%		29%	29%		
		Unknown	1%	1%		1%	1%		
Household income		$100K+	13%	13%	0.1206	13%	14%	0.0366	
		$40K - $49K	8%	9%		8%	8%		
		$50K - $59K	8%	8%		8%	8%		
		$60K - $74K	9%	9%		9%	9%		
		$75K - $99K	11%	11%		11%	11%		
		<$40K	35%	31%		34%	33%		
		Unknown	15%	18%		16%	16%		
Geographic location		Mountain	7%	8%	0.1574	7%	7%	0.0316	
		Northeast	18%	15%		17%	18%		
		Pacific	10%	13%		11%	11%		
		South Atlantic	25%	21%		24%	24%		
		Southern Midwest	14%	16%		14%	14%		
		Unknown	0%	0%		0%	0%		
		Upper Midwest	26%	27%		27%	27%		
Hypertension			83%	91%	0.2318	86%	86%	0.0051	
Hypercholesterolemia			75%	81%	0.1568	76%	77%	0.0083	
Chronic Kidney Disease		CKD	30%	45%	0.3136	34%	34%	0.0000	
		ESRD	0%	0%		0%	0%		
		Normal	70%	55%		66%	66%		
Ischemic heart disease			33%	46%	0.2684	37%	38%	0.0181	
Ischemic stroke			18%	43%	0.5656	25%	25%	0.0058	
Heart failure			18%	29%	0.2564	22%	22%	0.0096	
Peripheral arterial disease			21%	39%	0.3843	27%	27%	0.0110	
Diabetes Mellitus			37%	46%	0.1966	39%	39%	-0.0066	
Intracerebral Hemorrhage			1%	3%	0.1878	2%	2%	-0.0053	
Chronic Liver disease			1%	1%	0.0668	1%	1%	-0.0005	
Chronic Pulmonary Disease			14%	19%	0.1519	15%	15%	-0.0058	
Peripheral vascular disease			23%	41%	0.3889	29%	29%	0.0112	
Atrial fibrillation/flutter			19%	26%	0.1564	21%	22%	0.0072	
Congestive heart failure			19%	31%	0.2682	23%	22%	0.0100	
Myocardial infarction			9%	17%	0.2455	11%	11%	0.0049	
Arrhythmia			27%	42%	0.3048	32%	32%	0.0084	
Coronary Artery Bypass Graft			1%	1%	0.0772	1%	1%	0.0084	
Smoking			17%	26%	0.2684	20%	20%	0.0045	
Number of glaucoma med classes [mean (SD)]			0.7 (1.0)	0.6 (0.9)	-0.1568	0.7 (0.9)	0.7 (1.0)	-0.0071	
Number of glaucoma surgeries [mean (SD)]			0.1 (0.3)	0.0 (0.3)	-0.0589	0.1 (0.3)	0.1 (0.3)	-0.0006	
Number of health care visits [mean (SD)]			9.7 (7.1)	10.7 (7.9)	0.1259	10.0 (7.3)	10.2 (7.6)	0.0253	

**Table 2:** Baseline characteristics of neovascular age-related macular degeneration patients with and without dementia

Comparison of baseline characteristics between cases and contols in AMD cohort									
Characteristic			Before Weighting			After Weighting			
			No Dementia (n = 1908)	Dementia (n = 932)	Standardized mean difference	No Dementia (n = 1908)	Dementia (n = 932)	Controls (n = 1908)	
Age [mean (SD)]			85.0 (3.9)	84.6 (4.1)	-0.1034	85.1 (3.9)	84.7 (4.0)	-0.0842	
Gender	Female		71%	70%	-0.0092	71%	71%	-0.0032	
	Male		29%	30%		29%	29%		
Race	Black		1%	1%	0.1421	1%	1%	0.0000	
	Hispanic		1%	1%		1%	1%		
	White		99%	98%		99%	99%		
Education level	Bachelor Degree Plus		18%	19%	0.0994	18%	18%	0.0000	
	Less than Bachelor Degree		57%	60%		58%	58%		
	Less than or equal to HS Diploma		25%	21%		24%	24%		
	Unknown		0%	0%		0%	0%		
Household income	$100K+		10%	10%	0.2691	10%	10%	0.0536	
	$40K - $49K		7%	8%		7%	7%		
	$50K - $59K		6%	6%		6%	6%		
	$60K - $74K		6%	9%		7%	7%		
	$75K - $99K		8%	8%		8%	8%		
	<$40K		37%	27%		34%	35%		
	Unknown		25%	34%		28%	29%		
Geographic location	Mountain		14%	11%	0.1529	13%	14%	0.0577	
	Northeast		16%	16%		16%	16%		
	Pacific		12%	12%		12%	11%		
	South Atlantic		19%	17%		19%	18%		
	Southern Midwest		9%	9%		8%	9%		
	Unknown		0%	0%		0%	0%		
	Upper Midwest		29%	36%		32%	32%		
Hypertension			84%	89%	0.1544	86%	86%	0.0123	
Hypercholesterolemia			65%	71%	0.1366	67%	69%	0.0454	
Chronic Kidney Disease		CKD	25%	36%	0.2406	28%	27%	0.0224	
		ESRD	0%	0%		0%	0%		
		No disease	75%	64%		72%	73%		
Ischemic heart disease			39%	46%	0.1427	41%	43%	0.0367	
Ischemic stroke			26%	52%	0.5369	35%	35%	0.0117	
Heart failure			27%	34%	0.1355	30%	30%	-0.0087	
Peripheral arterial disease			28%	41%	0.2957	32%	31%	-0.0144	
Diabetes Mellitus			28%	31%	0.0465	29%	29%	-0.0005	
Intracerebral Hemorrhage			1%	3%	0.1743	2%	2%	-0.0058	
Chronic Liver disease			0%	0%	0.0285	0%	0%	-0.0085	
Chronic Pulmonary Disease			7%	9%	0.0861	7%	7%	-0.0026	
Peripheral vascular disease			31%	45%	0.3091	35%	35%	-0.0119	
Atrial fibrillation/flutter			25%	31%	0.1379	27%	27%	-0.0027	
Congestive heart failure			29%	35%	0.1182	32%	31%	-0.0115	
Myocardial infarction			13%	21%	0.2197	16%	16%	0.0045	
Arrhythmia			40%	50%	0.2	44%	44%	0.0056	
Coronary Artery Bypass Graft			0%	0%	0.0389	0%	0%	0.0148	
Smoking			11%	19%	0.2168	14%	13%	-0.0058	
Number of health care visits [mean (SD)]			9.9 (7.7)	9.5 (7.6)	-0.0439	9.8 (7.5)	9.9 (7.1)	0.0137	

*SD* standard deviation

The number of eye care visits and associated diagnostic tests and procedures in the first year after the index date for POAG and nAMD are presented in Table 3. POAG patients with dementia averaged 1.48 (SD ± 1.86) eye care visits in the first year after diagnosis compared with 2.15 (SD ± 2.15) in POAG patients without dementia. The average number of HVF (0.31 vs. 0.53), OCTs (0.23 vs. 0.39) and average days of prescription coverage (59.94 vs. 84.54) were also lower in POAG patients with dementia compared to those without. In IPTW Poisson regression models, POAG patients with dementia had a reduced rate of eye care visits (RR 0.76, 95% CI: 0.75–0.77, *p* < 0.0001) compared to those without dementia. The disparity between dementia and control patients was more pronounced for the rate of visual fields (RR 0.66, 95% CI: 0.62–0.68, *p* < 0.0001) and OCT (RR 0.67, 95% CI: 0.64–0.69, *p* < 0.0001) testing utilization. POAG patients with dementia had a lower rate of prescription coverage for intraocular pressure-lowering medications than controls (RR 0.83, 95% CI: 0.83–0.83, *p* < 0.0001).

**Table 3:** Annual measures of eye care utilization among primary open-angle glaucoma and neovascular age-related macular degeneration patients with and without dementia

		Dementia (*n* = 13,182)	No dementia (*n* = 32,288)	Adjusted rate ratio* (95% CI)
POAG	Number of eye care visits (mean, [SD])	1.48 (1.86)	2.15 (2.05)	0.76 (0.75, 0.77)
	Number of visual field tests (mean, [SD])	0.31 (0.56)	0.53 (0.65)	0.66 (0.63, 0.68)
	Number of OCTs (mean, [SD])	0.23 (0.48)	0.39 (0.58)	0.67 (0.64,0.69)
	Days of prescription coverage (mean, [SD])	59.94 (96.37)	84.54 (110.82)	0.83 (0.83, 0.83)
		Dementia (*n* = 932)	No Dementia (*n* = 1908)	Adjusted Rate Ratio* (95% CI)
nAMD	Number of eye care visits (mean, [SD])	1.33 (2.12)	2.07 (2.58)	0.74 (0.70, 0.79)
	Number of anti-VEGF injections (mean, [SD])	0.64 (2.16)	1.19 (2.71)	0.64 (0.58, 0.69)

*POAG* primary open angle glaucoma, *AMD* age-related macular degeneration, *SD* standard deviation

*Adjusted using inverse probability treatment weighting (IPTW). All *p*-values < 0.0001

nAMD patients with dementia were also seen less often by eye care providers (1.33 vs. 2.07 visits, RR 0.74, 95% CI: 0.70–0.79, *p* < 0.0001) and received fewer intravitreal anti-VEGF injections (0.64 vs. 1.19 injections; RR 0.64, 95% CI: 0.58–0.69, *p* < 0.0001) than nAMD patients without dementia. Similar findings were observed in our secondary analysis in which dementia cases were identified using a combination of diagnosis codes and dementia-related prescriptions (Supplemental Table 2).

## Discussion

In this study of over 45,000 older adults in the U.S., we examined healthcare utilization patterns for POAG and nAMD among persons newly diagnosed with dementia. We found that compared to those without dementia, POAG and nAMD patients with dementia obtained less eye care and received less monitoring and treatment of their disease. The rate of reduction in care was fairly consistent across both disease cohorts, with patients having dementia averaging about 75% of the number of visits for non-dementia patients. In-office testing and treatment were also similarly reduced across both diseases, with dementia patients receiving about two thirds of the testing and injections that patients without dementia received. Prescription drug coverage, while reduced, was not as impacted, with dementia patients receiving only 17% less coverage. This may be due to the ease of obtaining prescription medications without the requirement of an office visit. It is possible that reduced POAG medication use in patients with dementia was due to an increase in the use of selective laser trabeculoplasty (SLT) or argon laser trabeculoplasty (ALT), but this is unlikely because these procedures would not impact the need for disease monitoring within the first year of diagnosis and treatment, and we adjusted our analyses for baseline glaucoma surgical history. While our study is not determinative of a causal relationship between dementia and reduced treatment rate, these findings suggest that persons living with dementia may be vulnerable to gaps in ophthalmic care.

Our results are consistent with previous studies showing that persons living with dementia are less likely to be seen by an ophthalmologist or receive cataract surgery [9–11]. Disparities in anti-VEGF treatment for AMD have been observed on the basis of race [15], but to our knowledge, the effect of dementia on healthcare utilization for nAMD has not previously been examined. This growing body of literature indicates that persons living with dementia are vulnerable to gaps in ophthalmic care. While our study was not equipped to identify the specific reasons why patients with dementia were less likely to be seen by an ophthalmologist, potential reasons include difficulty making, remembering, and arranging transportation to appointments, especially if caregiver support is limited. Hospitalizations and other comorbidities may also limit the continuity of outpatient ophthalmic care. Visual impairment is an important determinant of quality of life and is associated with progression and neuropsychiatric complications of dementia [5–7], so it is possible that promoting eye care utilization in this population may improve functional outcomes. However, while there is preliminary evidence that regular eye care encounters and cataract surgery are associated with a lower incidence of dementia and functional decline [16, 17], the effects of eye care utilization on outcomes among those already living with dementia have not been examined.

Of note, not all underutilization of ophthalmic care is necessarily avoidable or harmful. We found that POAG patients with dementia were 34% less likely to have a visual field test than those without dementia, but because of the cognitive demands associated with maintaining fixation and responding quickly and accurately, visual field tests may not be possible or produce reliable results in persons living with dementia [18]. If cognitive concerns were the primary reason that visual field tests were underutilized, we would have expected greater utilization of OCT, which is more rapid and relies less heavily on prolonged fixation and patient participation. However, interpreting retinal nerve fiber layer and ganglion cell layer thinning in the setting of comorbid glaucoma and dementia is challenging, as these changes are known to occur in neurodegenerative diseases such as Alzheimer’s disease independent of glaucoma [19, 20]. Most patients with POAG are asymptomatic in their early stages, and treatment is aimed at reducing the progression of visual field constriction over years or decades. For people with limited life expectancy due to advanced dementia, these treatments may not yield meaningful benefits if death is expected to occur before clinically significant visual impairment due to untreated glaucoma develops. However, such decisions would require a highly personalized assessment of life expectancy and glaucoma progression, and the clinical and health-economic implications of the de-escalation of ophthalmic care at the end of life have not been examined. In the case of nAMD, anti-VEGF injections have the potential to result in more immediate improvements in visual acuity, but these benefits must be weighed against the tradeoffs in quality of life associated with frequent ophthalmology visits and injection-related discomfort and logistical barriers such as ability to consent and availability of legal guardianship.

The results of this study must be considered within the limitations of its design. Due to the deidentified nature of administrative claims data, we lacked information about ocular disease severity such as visual acuity and OCT results, which are important determinants of visit frequency and treatment. The identification of ophthalmic diagnoses and healthcare utilization within administrative claims data is contingent upon insurance billing and reimbursement, so if patients paid for visits, procedures, or medications out of pocket, these would theoretically not be captured. However, this is very unlikely because the outcomes we examined are covered by insurance, and therefore unlikely for patients to ignore their insurance benefit to pay more out of pocket for these services. Another limitation is the reliance on ICD codes to identify dementia, as dementia is frequently underdiagnosed in clinical practice [21]. To counter this, we tested a more expansive definition of dementia, which did not alter the results of the study. Moreover, if the control population contained persons living with dementia who were misclassified as controls, we would expect this to bias results toward the null, suggesting that our results are an underestimate of the impact of dementia on eye care utilization for these disease states. Older adults in this dataset have private health insurance or are primarily covered through Medicare Advantage plans, and while we would expect similar findings in the Medicare fee-for-service (FFS) population, this is difficult to confirm due to known demographic and socioeconomic differences between FFS and Medicare Advantage enrollees. Finally, type 1 error is always a possibility, especially in the context of multiple hypothesis testing.

We found that being diagnosed with dementia leads to lower utilization of eye care in the year following diagnosis. Although the rate of eye care visits was lower, the largest disparities in care were related to the ancillary testing utilization and treatment. Future studies should examine whether eye care underutilization is associated with worse visual, cognitive, and functional outcomes in people living with dementia. If this is the case, then efforts to promote access to eye care may have a positive public health impact in this population.

## Electronic Supplementary Material

Below is the link to the electronic supplementary material.

Supplementary Material 1
