# Supplementary material for: Disparities in glaucoma and macular degeneration healthcare utilization among persons living with dementia in the United States
Source: Graefes Arch Clin Exp Ophthalmol. 2024 Jul 12;262(12):3947–55. doi: 10.1007/s00417-024-06573-z (PMC11608381; doi:10.1007/s00417-024-06573-z)
Supplement: Supplementary file 1 — Supplementary Material 1 [file 417_2024_6573_MOESM1_ESM.docx]

Supplemental Table 1: Diagnosis, procedure, and provider codes used in this study

| **Diagnosis Category** | **ICD-9 Codes** | **ICD-10 Codes** |
| --- | --- | --- |
| Primary Open Angle Glaucoma | 365.10, 365.11, 365.12, 365.15, 365.7x | H40.10x, H40.11x, H40.12x, H40.15x |
| Non-POAG Diagnosis (excluded) | 365.2, 365.3, 365.4, 365.5, 365.6, 365.8 | H40.13x, H40.14x, H40.2x, H40.3x, H40.4x, H40.5x, H40.6x H40.8x, H40.9s |
| Exudative (neovascular) AMD | 362.52 | H35.32xx |
| Diseases that could use anti-VEGF treatments from AMD cohort (excluded) | 362.02, 364.42, 365.63, 365.89, 362.15, 362.16, 362.29, 362.07, 362.82, 362.83, 362.53, 379.23, 361.81, 361.9, 365.53, 282.6x, 362.2x, 362.3x, 362.4x | E08-E13. with .35x, E08-E13. with .311, .321, .331, .341, .37x, H43.1x, H33.4x, H40.5x, H40.89x, D57.x, H35.x, H35.35x, H35.81, H35.89, H34.8x, H34.0x, H34.1x, H34.2x, H33.2x |
| Dementia | 3310, 33111, 33119, 3312, 3317, 2900, 29010, 29011, 29012, 29013, 29020, 29021, 2903, 29040, 29041, 29042, 29043, 2908, 2940, 29410, 29411, 29420, 29421, 33182, 33189 | F0150, F0151, F0280, F0281, F0390, F0391, F04, G300, G301, G308, G309, G3101, G3109, G3183, G311, G312, R4181 |
| **Outcomes** | **CPT Code** | **PROVCAT Code** |
| Number of eye care visits after the index date in the first year (Glaucoma) | 99201, 99202, 99203, 99205, 99211, 99212, 99213, 99214, 99215, 99241, 99242, 99243, 99244, 99245, 92002, 92004, 92012, 92014 | 0116-0118, 0119-0121, 0633-0640, 1145, 1222, 1243, 1254, 1283, 1316, 1326, 1399, 1689, 1706, 1739, 1853, 1867, 1934, 1941, 2003, 2083, 2098, 2120, 2121, 2122, 2123, 2266, 2269, 2328, 2350, 2383, 2386, 2443, 2601, 2612, 2621, 2699, 2857, 2869, 2929, 2941, 3006, 3039, 3126, 3184, 3379, 3380, 3520, 3526, 3615, 3763, 3876, 3920, 3956, 4066, 4121 |
| Number of visual fields within the first year after the index date | 92081, 92082, 92083 |  |
| Number of OCT's performed within the first year after the index date | 92133 |  |
| Treatment of AMD | 67028 |  |
| Number of eye care visits after the index date in the first year (AMD) | 99202, 99203, 99205, 99211, 99212, 99213, 99214, 99215, 99241, 99242, 99243, 99244, 99245, 92002, 92004, 92012, 92014 | 0116-0118, 0119-0121, 0633-0640, 1145, 1222, 1243, 1254, 1283, 1316, 1326, 1399, 1689, 1706, 1739, 1853, 1867, 1934, 1941, 2003, 2083, 2098, 2120, 2121, 2122, 2123, 2266, 2269, 2328, 2350, 2383, 2386, 2443, 2601, 2612, 2621, 2699, 2857, 2869, 2929, 2941, 3006, 3039, 3126, 3184, 3379, 3380, 3520, 3526, 3615, 3763, 3876, 3920, 3956, 4066, 4121 |

Supplemental Table 2: Results from sensitivity analysis using both diagnosis codes and prescriptions to identify dementia patients with primary open-angle glaucoma and age-related macular degeneration patients (vs. diagnosis codes only)

|  |  | Dementia  (n=13552) | No Dementia  (n=33050) | Adjusted Rate Ratio (95% CI) |
| --- | --- | --- | --- | --- |
| POAG | Number of eye care visits (mean, [SD]) | 1.53 (1.87) | 2.15 (2.04) | 0.78  (0.77, 0.79) |
|  | Number of visual field tests (mean, [SD]) | 0.33 (0.58) | 0.53 (0.65) | 0.70  (0.68, 0.72) |
|  | Number of OCT (mean, [SD]) | 0.24 (0.49) | 0.38 (0.58) | 0.70  (0.68, 0.72) |
|  | Days of prescription coverage (mean, [SD]) | 61.47 (97.43) | 84.29 (110.73) | 0.85  (0.84-0.85) |
|  |  |  |  |  |
|  |  | Dementia  (n=912) | No Dementia  (1891) | Adjusted Rate Ratio (95% CI) |
| AMD | Number of eye care visits (mean, [SD]) | 1.41 (2.28) | 2.09 (2.60) | 0.75  (0.70, 0.79) |
|  | Number of anti-VEGF injections (mean, [SD]) | 0.66 (2.26) | 1.15 (2.69) | 0.62  (0.57, 0.68) |

POAG, primary open angle glaucoma; AMD, age-related macular degeneration; SD, standard deviation

*Adjusted using inverse probability treatment weighting (IPTW). All p-values <0.0001
